# The combined role of obesity and depressive symptoms in the association with ischaemic heart disease and its subtypes

**DOI:** 10.1038/s41598-022-18457-5

**Published:** 2022-08-24

**Authors:** Shuo Liu, Jia Luo, Tianhao Zhang, Dongfeng Zhang, Hua Zhang

**Affiliations:** 1 Department of Epidemiology and Health Statistics, Qingdao University Medical College, Qingdao, People’s Republic of China; 2 Department of Epidemiology and Health Statistics, The School of Public Health of Qingdao University, The College of Public Health of Qingdao University, 308 Ningxia Road, Qingdao, 266071 Shandong People’s Republic of China; 3 Municipal Centre of Disease Control and Prevention of Qingdao, Qingdao, 266034 Shandong People’s Republic of China

**Keywords:** Cardiology, Neurology, Risk factors

## Abstract

This cross-sectional study aimed to explore the combined effects of depression and obesity on ischemic heart disease and its subtypes. Data from the National Health and Nutrition Examination Survey 2007–2018 were used. A total of 29,050 participants aged 20 years or older were included in the analyses. Logistic regression models and restricted cubic spline models were applied to evaluate the associations between depression symptom and ischemic heart disease. There were significant correlations between depressive symptoms and ischemic heart disease [OR and 95% CI 2.44 (1.91, 3.10)] and its subtypes: coronary heart disease [2.32 (1.67, 3.23)], heart attack [2.18 (1.71, 2.78)], and angina [2.72 (1.96, 3.79)].The synergistic effects of depression with obesity (BMI ≥ 30) and central obesity (waist ≥ 102/88 cm for men/women) on ischemic heart disease were estimated and expressed using the relative excess risk due to interaction (RERI) and the attributable proportion due to interaction (AP). The RERI and AP with 95% CIs of depression and central obesity for ischemic heart disease were 1.10 (0.01, 2.19) and 0.35 (0.06, 0.64). When we analysed the other three subtypes of ischemic heart disease, we only found depressive symptoms and central obesity could have a meaningful synergistic effect on heart attack (RERI: 0.84 (− 0.28, 1.96) AP: (0.31 (0.00, 0.69)).

## Introduction

According to WHO’s Global Health Estimates, ischaemic heart disease is the leading cause of death globally, and it is responsible for 16% of the world’s total deaths^1^. On the basis of some epidemiological studies, the occurrence of ischemic heart disease is related to many factors, such as smoking^2^, high consumption of fatty acids^3^, and a lack of exercise^4^. In some recent studies, several social and psychological factors have also been reported to contribute to ischaemic heart disease^5–13^.

Depression is a common mental illness. In recent years, more than 300 million people worldwide suffer from depression each year^14^. Some pathological studies have found that depression can activate inflammatory pathways by increasing proinflammatory factors^15^. Moreover, depression is also associated with changes in platelet function and impaired endothelial function^16,17^. All of these factors may contribute to the onset of ischaemic heart disease. Some epidemiological studies have revealed the relationship between depression and ischaemic heart disease^5,18,19^. A meta-analysis, which combined longitudinal evidence from 21 studies involving more than 120,000 subjects, concluded that depression increased the risk of cardiovascular disease by 80–90%^20^. However, most studies incompletely adjusted for conventional risk factors, and the adjusted risk estimates might be exaggerated.

Over the past two decades, obesity has become a public health problem worldwide, affecting children and adults alike. Many studies have confirmed that obesity is an independent risk factor for cardiovascular disease. Adipokines released by adipose tissue can induce endothelial dysfunction, systemic inflammation, and insulin resistance, all of which can increase the risk of atherosclerosis^21–23^.

From what has been discussed above, depressive symptoms and obesity share pathophysiological pathways that include exacerbating inflammatory responses and endothelial dysfunction, which may contribute to the formation of atherosclerosis^23,24^. Therefore, a possible mechanism in which depression and obesity may mutually promote their respective pathophysiological mechanisms has been proposed to contribute to the development of ischaemic heart disease^25–27^. According to speculation, it is suggested that there may be a synergistic effect between depressive symptoms and obesity. To date, limited studies have explored the synergistic effect between depressive symptoms and obesity, and the results have been inconsistent. Few studies have explored this combined effect on different types of ischaemic heart disease. A study of American adults by Brittanny et al. found significant interactions between obesity and depression^28^. However, in the study by Elisabeth et al., the interaction between depressive symptoms and obesity was reversed and not statistically significant^29^. These inconsistent findings highlight the need for continued research into the combined role of depression and obesity in ischaemic heart disease. Therefore, in this study, we explored the synergistic effects of depressive symptoms and obesity on ischaemic heart disease.

## Materials and methods

### Study population

The NHANES is a population-based study in the United States in which researchers use a complex, layered, multistage, probabilistic sampling method to select a representative population. Since 1999, data have been collected in a biennial cycle. It includes a personal interview and a standardized medical examination^30^. The protocol has been approved by the Ethics Review Committee of the National Center for Health Statistics and written informed consent is obtained from participants^31^. The methods used in this article were in accordance with the relevant guidelines and regulations.

**Figure 1:**
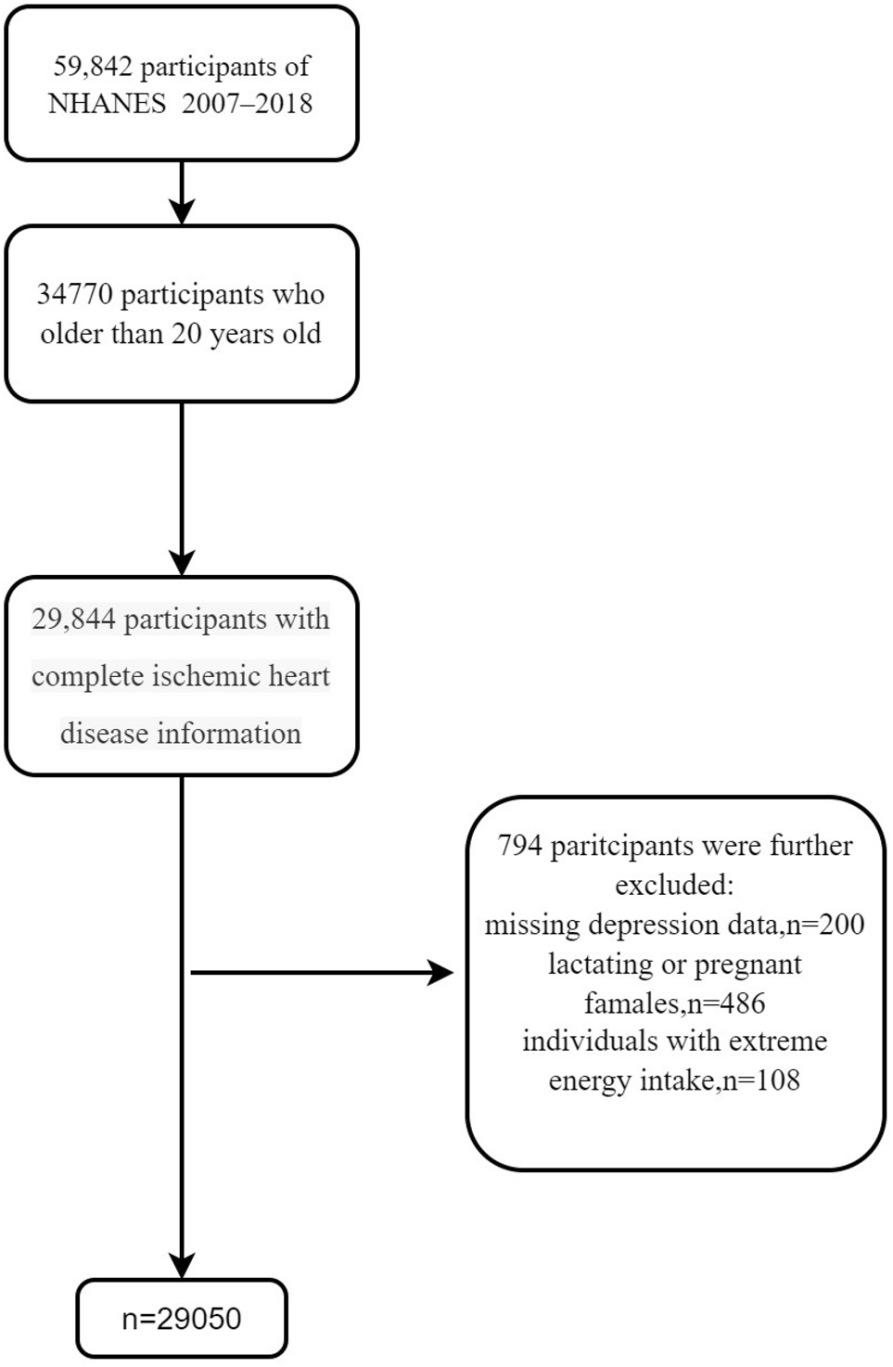
Flow diagram of the selection of eligible participants, NHANES 2007–2018.

A total of 59,842 participants in the NHANES during 2007–2018 were included in our study. We selected participants aged 20 years or older for the study, with a total of 34,470 people. From these participants, we excluded those with incomplete information for either the depression questionnaire (n = 4926) or the ischaemic heart disease questionnaire (n = 200). Females who were pregnant (n = 274) or breastfeeding (n = 212) were also excluded. In addition, we excluded individuals with extreme energy intake (500 or 5000 kcal/day for women and 500 or 8000 kcal/day for men) (n = 108). Ultimately, the study included 29,050 participants (14,509 males and 14,541 females) (Fig. 1).

### Depressive symptom assessment

In the NHANES, depressive symptom status is assessed using the Patient Health Questionnaire (PHQ-9). The questionnaire assesses the frequency of nine depressive symptoms in the past 2 weeks^32^. The total scores for the PHQ-9 range from 0 to 27, where each question is scored on a 4-point Likert scale: 0 = "none at all," 1 = "a few days," 2 = "more than half the time," and 3 = "almost every day." We used a score ≥ 10 as the standard for depressive symptoms. The sensitivity and specificity of the questionnaire for major depression are 88% and 88%, respectively^33^.

### Obesity assessment

Waist circumferences (WC) were measured during minimal respiration to the nearest 0.1 cm at the level of the iliac crest^34^. BMI was calculated as weight in kilograms divided by the square of height in meters. Central obesity was defined as a WC of ≥ 102 cm for males and ≥ 88 cm for females according to the American Heart Association’s (AHA) definition^35^. We also used a BMI ≥ 30 kg/m^2^ to indicate obesity based on the WHO’s definition of obesity^36^.

### Ischemic heart diseases outcomes

We used ischaemic heart disease as the primary outcome. Ischaemic heart disease was defined if one of our prespecified secondary outcomes (coronary heart disease, angina and heart attack) occurred^37^. Secondary outcomes were assessed during personal interviews using a standardized health status questionnaire in which participants were asked "Has a doctor or other health professional ever told you that you have coronary artery disease/angina/heart attack?". The average interval between individual interviews and mobile test centre (MTC) tests was 2 weeks.

### Covariates

By assessing the association between depression symptoms and ischaemic heart disease, covariates were selected and controlled. We selected covariates for age, sex, ethical groups, education level, income level, work activities, recreational activities, smoking status, alcohol use, diabetes, hypertension, and BMI. The selection of covariates was based on similar studies^28,29^. These covariates were used when we used logistic regression to explore the relationship between depressive symptoms and ischaemic heart disease. When we calculated the RERIs and APs, we omitted BMI from the covariables. See Table S1 for a detailed description of the related covariates.

### Statistical analyses

We used STATA 15.0, SPSS 26.0, and R programming language 4.0.3 for our analyses. The NHANES guidelines recommend that multiple cycles be combined for estimation to increase the sample size. To explain the complexity of the sampling design, appropriate sampling weights, original sampling units, and stratigraphic information were selected for analysis. When combining two or more consecutive periods, a new sample weight was constructed before any analysis was initiated.

Normally distributed variables are represented by the mean ± standard deviation, nonnormally distributed variables are represented by the median (quartile range), and categorical variables are represented by numbers (percentages). According to the Kolmogorov‒Smirnov normality test, scores on the depression scale had a nonnormal distribution and are described using the median (quartile range). For continuous variables, if the variables were normally distributed, we used Student’s t test to compare the average level of continuous variables between participants with and without depressive symptoms. Otherwise, nonparametric tests were used. In addition, the chi-square test was used to compare the percentage of categorical variables between groups. Univariate and multivariate logistic regression analyses were used to assess the association between depressive symptoms and the risk of ischaemic heart disease and its subtypes, including coronary heart disease, myocardial infarction, and angina pectoris. Depressive symptoms and depression scores were analysed in the model as categorical variables and continuous variables, respectively. Model 1 adjusted for age and sex; Model 2 adjusted for all covariates. Odds ratios (ORs) and 95% confidence intervals (CIs) were calculated. The dose‒response relationship was evaluated by a restricted cubic spline model, with three segments located in the 5th, 50th, and 95th percentiles of the scale. A PHQ-9 score of 0 was used as the reference group. In addition, we performed a series of analyses stratified by age (45 years, 45–64 years, and ≥ 65 years), sex (male and female), obesity (BMI < 30, BMI ≥ 30), central obesity (WC < 102/88 cm for males/females, WC ≥ 102/88 cm for males/females) and ethnic groups (Mexican American, Other Hispanic, Non-Hispanic White, Non-Hispanic Black, Other Race). Considering the relationship between menopause and depression and cardiovascular disease, we further stratified women into menopausal and nonmenopausal women for analysis^38,39^.

The synergistic effects of depression with obesity and central obesity on ischaemic heart disease were estimated and expressed using the relative excess risk due to interaction (RERI) and the attributable proportion due to interaction (AP), adjusting for potential confounders. The formula for the RERI is as follows: RR11 − RR01 − RR10 + 1. The AP is calculated as RERI/RR11. RR11, RR10, and RR01 represent the logistic regression adjusted estimated RR of the covariates of depression and obesity, depression but not obesity, and obesity but not depression, respectively. Control subjects were neither depressed nor obese (RR00). An RERI and AP greater than 0 indicated a synergistic effect between depression and BMI (or waist circumference). Hosmer and Lemeshow's methods were used to calculate the 95% CIs for the RERI and AP^40^.

### Sensitivity analysis

To make our results more representative, we performed two sensitivity analyses. First, there was an absence of 14.3% of the depression score scale data in our analysis. Therefore, we used the multi-interpolation method to interpolate the dataset lacking depression data. Missing data were filled in using Monte Carlo methods. Average and exponential operations were performed on the coefficients of the estimated dataset to calculate proportional risk. The standard error and p value were calculated to reflect the uncertainty caused by missing values and multiple interpolations^41^. We performed the above calculations using the MICE package of the R language^42^. Second, in cancer patients, depressive symptoms are more prevalent, and increased viscosity increases the risk of cardiovascular disease^43,44^. Therefore, we excluded people with cancer for logistic regression analysis in Model 2.

### Ethics approval

Public release data in NHANES was used for our research data. The protocol is approved by the Ethics Review Committee of the National Center for Health Statistics. The ethics code for each wave data is provided on the NHANES website. The NCHS IRB/ERB Protocol Numbers we used for the data are as follows: Continuation of Protocol #2005-06, Continuation of Protocol #2005-06, Protocol #2011-17, Continuation of Protocol #2011-17, Continuation of Protocol #2011-17, Protocol #2018-01 (Effective beginning October 26, 2017) and Continuation of Protocol #2011-17 (Effective through October 26, 2017).

## Results

The characteristics of the 29,050 included participants by depressive symptoms status are depicted in Table S2. The prevalence of depression symptoms (PHQ-9 score ≥ 10) was 9.07%. There were significant differences in demographic characteristics between the depressive symptoms group and the nondepressive symptoms group. People with depressive symptoms were more likely to be female, younger, smokers, drinkers, and obese and to have hypertension, diabetes, less education, lower family income, lower work activity and recreational activity, lower total energy intake, and higher caffeine intake. In addition, the depressive symptoms group had a higher percentage of individuals with ischaemic heart disease or its subtypes.

Table 1 shows the results of the logistic regression analyses. Depressive symptoms were positively associated with the risk of ischaemic heart disease, coronary heart disease, angina, and heart attack based on crude odds ratios (ORs) and 95% confidence intervals (CIs) for depressive symptoms. When the PHQ scores were analysed in the logistic regression as a continuous variable, the results were still significant. After adjustment for age and sex (Model 1), the results were similar to those of the crude model. After further adjustment in Model 2, there was a significant correlation between depressive symptoms and ischaemia and its subtypes (coronary heart diseases, heart attack and angina), with multivariate-adjusted ORs (95% CIs) of 2.44 (1.91, 3.10), 2.32 (1.67, 3.23), 2.18 (1.71, 2.78), and 2.72 (1.96, 3.79), respectively. The results are shown using a forest map (Fig. S1).

**Table 1:** Weighted odds ratios (95% confidence intervals) of ischemic heart disease coronary heart disease heart attack and anginas across depressive symptoms, NHANES 2007–2018 (N = 29,050).

	Crude (N = 29,050)	Model 1 (N = 29,050)	Model 2 (N = 22,598)
**Ischemic heart disease**			
OR (95% CI)	2.10 (1.75, 2.53)	3.01 (2.46, 3.68)	2.44 (1.91, 3.10)
P-value	< 0.001	< 0.001	< 0.001
**Coronary heart disease**			
OR (95% CI)	1.91 (1.49, 2.46)	2.80 (2.12, 3.69)	2.32 (1.67, 3.23)
P-value	< 0.001	< 0.001	< 0.001
**Heart attack**			
OR (95% CI)	2.13 (1.72, 2.66)	2.93 (2.35, 3.66)	2.18 (1.71, 2.78)
P-value	< 0.001	< 0.001	< 0.001
**Angina**			
OR (95% CI)	2.63 (2.01, 3.45)	3.31 (2.49, 4.40)	2.72 (1.96, 3.79)
P-value	< 0.001	< 0.001	< 0.001

^a^Calculated using binary logistic regression. Model 1 adjusted for age, gender. Model 2 adjusted for age, gender, ethical groups, educational level, household income, caffeine intake, total energy intake, smoking, alcohol consumption, work activity, recreational activity, diabetes, hypertension and BMI.

Table S3 shows the estimated associations between depressive symptoms and ischaemic heart disease and its subtypes stratified by age, sex, obesity, and central obesity. In sex stratification, the positive associations between ischaemic disease and depressive symptoms were significant in both the male and female groups. Then, when the female group was further subdivided, we found that depression remained a significant risk factor for ischaemic heart disease in nonmenopausal women, whereas the risk effect of depression was not significant for ischaemic heart disease in menopausal women. In age-stratified analysis, we found no meaningful association between depressive symptoms and coronary heart disease in the group of participants aged 20–39 years. In the other age groups, depressive symptoms were positively associated with ischaemic heart disease and its subtypes. In analyses stratified by obesity and central obesity, depressive symptoms were significantly associated with risk of ischaemic heart disease and its subtypes in all levels. Table S4 shows the results of the racially stratified analysis. Depressive symptoms were significant risk factors for ischaemic heart disease in Mexican Americans, Non-Hispanic Whites, and Non-Hispanic Blacks. However, we did not find this relationship with other Hispanics and other races.

Table 2 shows the combined effect of depression and obesity (BMI ≥ 30) and central obesity (WC ≥ 102/88 cm for males/females) on ischaemic heart disease. The combined effect of depression and central obesity was significantly greater than the sums of the individual effects. Compared with the reference group, the OR (95% CI) of only central obesity was 1.16 (0.93, 1.46), and the OR (95% CI) of only depression was 1.88 (1.24, 2.88), and 3.15 (2.32, 4.48) for both central obesity and depression. The RERI and AP with 95% CIs were 1.10 (0.01, 2.19) and 0.35 (0.06, 0.64) for depressive symptoms and central obesity. However, the additive interaction between depression and obesity was not significant. The RERI and AP were 0.90 (− 0.31, 2.12) and 0.26 (− 0.03, 0.55), respectively. Table S5 shows the synergistic effect of depression and obesity on the secondary outcomes, including the three types of ischaemic heart disease. Depressive symptoms and obesity were not significant for all three subtypes of ischaemic heart disease. Depressive symptoms and central obesity could have a meaningful synergistic effect on heart attack. The RERI and AP were 0.84 (− 0.28, 1.96) and 0.31 (0.00, 0.69), respectively. The synergistic effect was not significant when coronary heart disease and angina pectoris were included as the outcome variables.

**Table 2:** Synergic effect of depression and obesity on ischemic heart disease incidence, NHANES 2007–2018 (*N* = 29,050).

	Incidence (%)	OR (95% CI)	RERI (95% CI)	AP (95% CI)
**BMI category**				
BMI < 30.0 kg/m^2^ & Depression−	55.92	1	0.90 (− 0.31, 2.12)	0.26 (− 0.03, 0.55)
BMI ≥ 30.0 kg/m^2^ & Depression−	34.91	1.33 (1.13, 1.57)		
BMI < 30.0 kg/m^2^ & Depression+	4.48	2.21 (1.59, 3.09)		
BMI ≥ 30.0 kg/m^2^ & Depression+	4.69	3.49 (2.57, 4.73)		
**Waist category**				
Central obesity− & Depression−	38.37	1	1.10 (0.01, 2.19)*	0.35 (0.06, 0.64)**
Central obesity+ & Depression−	55.46	1.16 (0.93, 1.46)		
Central obesity− & Depression+	2.78	1.88 (1.24, 2.85)		
Central obesity+ & Depression+	6.39	3.15 (2.32, 4.48)		

The model adjusted for age, gender, ethical groups, educational level, household income, caffeine intake, total energy intake, smoking, alcohol consumption, work activity, recreational activity diabetes and hypertension.

*P < 0.05.

**P < 0.01.

**Figure 2:**
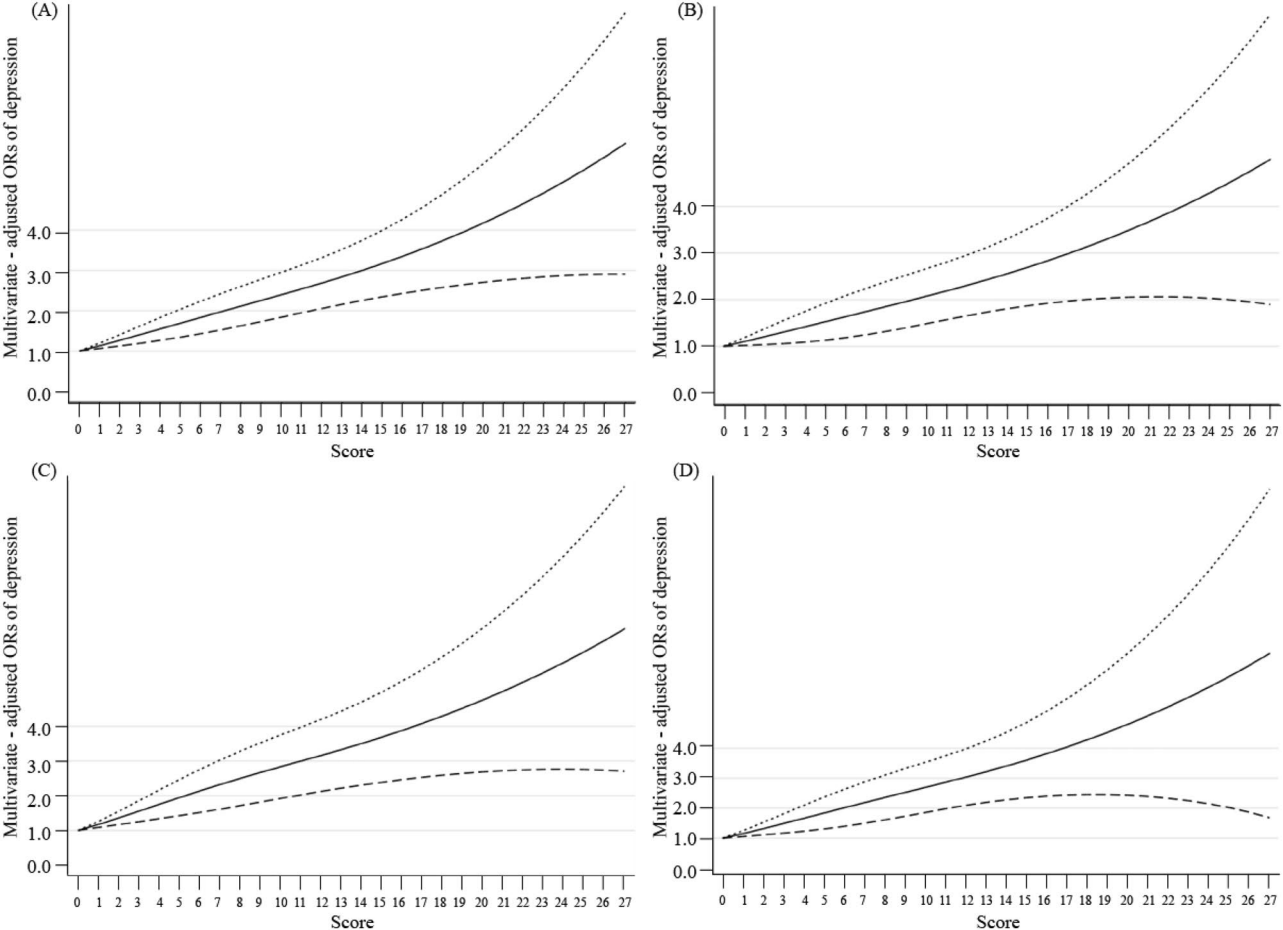
The dose–response relationships between PHQ scores and ischemic heart disease, coronary heart disease, heart attack, and angina pectoris. The solid line and dashed line represent the estimated ORs and its 95% CI. *OR* odds ratio, *CI* confidence intervals. (**A**–**D**) The outcome variables are ischemic heart disease, coronary heart disease, heart attack, and angina pectoris, respectively.

The dose‒response relationships between PHQ-9 scores and ischaemic heart disease, coronary heart disease, heart attack, and angina pectoris are shown in Fig. 2. In the restricted cubic spline model, we found linear relationships between PHQ scores and the risk of ischaemic heart disease (P for linearity < 0.0001), coronary heart disease (P for linearity = 0.015), heart attack (P for linearity < 0.0001), and angina (P for linearity = 0.001).

Table S6 describes the results of the analysis after multiple interpolations. Depressive symptoms remained a significant risk factor for ischaemic heart disease after multiple interpolations of data from 4864 patients with the Missing Depression Scale. After we excluded cancer patients, the risk effect of depressive symptoms remained significant in Model 2 (Table S7).

## Discussion

In this study, we used data from 29,050 participants at least 20 years of age from the NHANES (2007–2018) database. We found that depressive symptoms were associated with a higher risk of ischaemic heart disease and its subtypes, including coronary heart disease, angina pectoris and heart attack. When we performed the dose‒response relationship analysis, we found linear relationships between the PHQ-9 score and ischaemic heart disease and its subtypes. Furthermore, we observed that the combined effect of depressive symptoms and central obesity increased the risk of ischaemic heart disease. When we analysed the other three subtypes of ischaemic heart disease, we found that the synergistic effect of depressive symptoms and central obesity on heart attack was significant.

To date, several epidemiological studies have reported the combined effect of depression and obesity on ischaemic heart disease. A 14-year follow-up MONICA–KORA Augsburg Cohort Study including 6239 individuals indicated that the interaction between obesity and depression had a positive effect on the incidence of coronary heart disease. The interaction term between depression and obesity had an effect value of 1.73 (95% CI 0.98–3.05)^45^. A cohort study detected the combined effects of three cardiovascular risk factors and lifelong depression on CVD events. The results pointed out the positive multiplicative interaction between BMI and depressive disorders was significant (p = 0.031)^28^. A finding of a study on longitudinal ageing in Amsterdam was inconsistent with ours. Interaction effects between depressive disorders and obesity were negative and not statistically significant (RERI (95% CI) − 0.45 (− 1.31 to 0.41))^29^.

The underlying mechanisms of the relationship between depression and obesity, and an increased risk of ischaemic heart disease have been discussed in depth elsewhere and include the following^23,24,46–57^. Depression and obesity may have overlapping pathophysiological pathways, so depression and obesity may amplify each other's pathophysiological mechanisms of atherosclerosis. The first pathway is the dysfunction of the endocrine system. Depression and obesity are frequently accompanied by a chronic release of glucocorticoids, which lead to a slower response to their typical anti-inflammatory effects over time, whereafter, increasing the risk of cardiovascular disease^24,46,47^. Second, increased levels of inflammatory markers may mediate the progression of ischaemic heart disease in individuals with depression or obesity^48,49^. Both depression and obesity are associated with increased C-reactive protein, tumour necrosis factor-α, interleukin-1, and interleukin-6 levels^23,24,50,51^. Apart from overlapping physiological pathways, treatment nonadherence pathways could perhaps be comprised too^52,53^. By means of interfering with or controlling the primary prevention of obesity, depression may amplify the effect of obesity on atherosclerosis. Finally, the increased risk of shared behaviours in both depressed and obese individuals may lead to a higher risk of adverse cardiovascular health outcomes, such as a decrease in physical activity. Physical inactivity is common among people with depression and obesity^54,55^. It could lead to a higher risk of ischaemic heart disease^56,57^.

Our results suggest that the interaction with depression was better in central obesity than in general obesity. Many studies have described obesity as a major risk factor for cardiovascular disease^58–60^. However, a growing number of studies have shown that BMI cannot accurately reflect body mass distribution, and central obesity may be a more important predictor of cardiovascular disease^61–64^. In a case‒control study conducted by Winter and colleagues, markers of abdominal obesity, including WC and related ratios, were found to be better predictors of stroke and cerebrovascular events than BMI^64^. Similarly, Lakka and colleagues found that abdominal obesity was an independent risk factor for coronary heart disease in middle-aged men, and was even more important than overall obesity^61^.

This study had several strengths. First, we analysed the combined effects of depressive symptoms and obesity on three different subtypes of ischaemic heart disease. In addition, in order to make our results more scientific, we included a large nationally representative sample of adults in the US, as well as included and adjusted for known potential risk factors for ischaemic heart disease. In addition, additive interactions, which are relevant to public health and provide insight into mechanistic forms of interaction rather than statistical interaction, were applied to explore the combined effects of depressive symptoms and obesity in our study^65^. However, a few limitations should be considered and resolved in future research. It was difficult to obtain the causal inferences only using the cross-sectional design. Nevertheless, cross-sectional studies are necessary to obtain clues as to the cause of a disease. This study provides theoretical basis for the next cohort study and clinical trial. In addition, this study might be subject to recall bias because certain data were based on self-reports. Although several potential confounding factors were controlled, we still cannot exclude the possibility of residual confounding caused by unmeasured confounding factors.

In conclusion, in this cross-sectional study, depressive symptoms were associated with a higher risk of ischaemic heart disease in adults in the US. There was also a synergistic effect between central obesity and depressive disorders. This manuscript mainly underscores the increased risk of ischaemic heart disease in patients with depressive symptoms that are concomitant with obesity. The purpose of this article was to improve the compliance of patients with depressive symptoms to maintain a healthy weight. It is suggested that mental health professionals should pay attention to patients' behavioural habits while treating mental illness, help patients maintain a healthy weight and prevent patients from becoming obese or underweight.

## Supplementary Information

Supplementary Information.

## Data Availability

The data that support the findings of this study are openly available from the National Health and Nutrition Examination Survey with the WEB LINK: https://www.cdc.gov/nchs/nhanes/index.htm.
