# Supplementary material for: The combined role of obesity and depressive symptoms in the association with ischaemic heart disease and its subtypes
Source: Sci Rep. 2022 Aug 24;12:14419. doi: 10.1038/s41598-022-18457-5 (PMC9402922; doi:10.1038/s41598-022-18457-5)
Supplement: Supplementary file 1 — Supplementary Information. [file 41598_2022_18457_MOESM1_ESM.pdf]

The combined role of obesity and depressive symptom in the association with ischemic heart disease and its subtypes

Shuo liu[a], Jia Luo[a], Tianhao Zhang[a], Dongfeng Zhang[a], Hua Zhang[b*]

[a] Department of Epidemiology and Health Statistics, Qingdao University Medical college, Qingdao, China.

[b] Department of Epidemiology and Health Statistics, The College of Public Health of Qingdao University, Qingdao, Shandong, 266071, China; Municipal Centre of Disease Control and Prevention of Qingdao, Qingdao, Shandong, 266034, China.

Electronic address: cdcwswjyk@qd.shandong.cn.

[*] Correspondence: Prof. Hua Zhang. Department of Epidemiology and Health Statistics, the School of Public Health of Qingdao University, 308 Ningxia Road, Qingdao, Shandong 266071, People's Republic of China.

E-mail address: qdcdcsq@163.com.

Tel: (+86) (0532) 85650292.

Figure S1

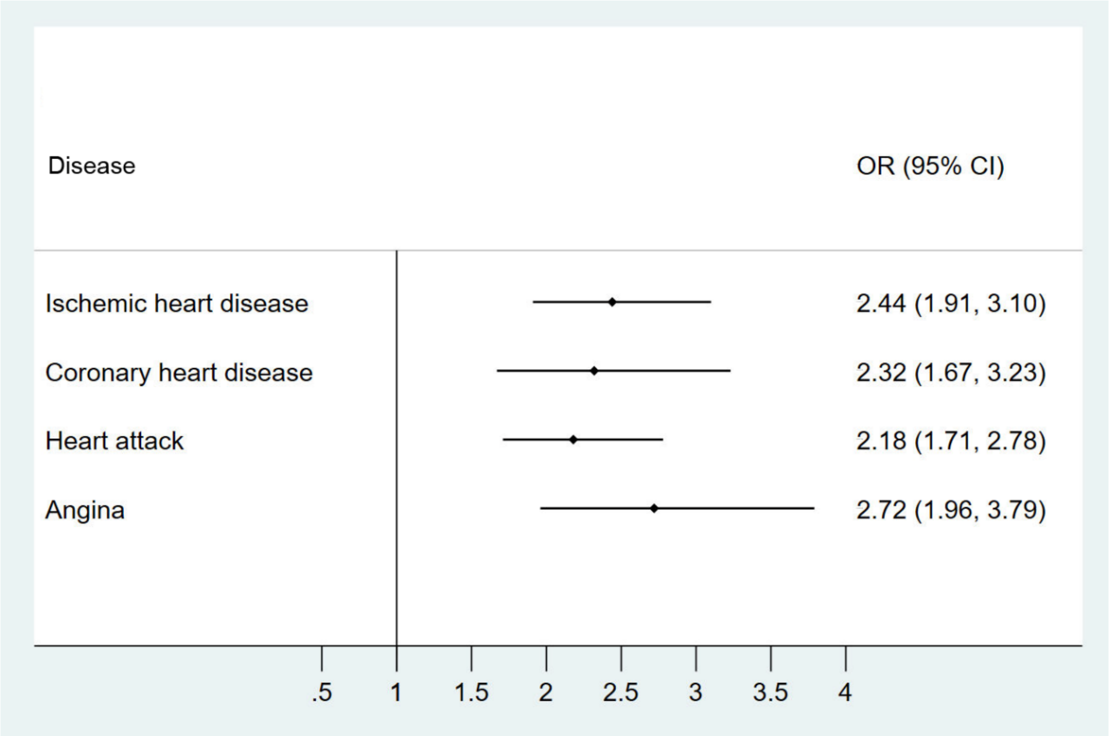

Figure S1: Forest plot depicts the ORs (95% CIs) of the association between depressive symptoms and ischemic heart disease and its subtypes.

Table S1. The classifications of covariates

Covariates	Classifications
**Gender**	Female; Male
**Age (year)**	Continuous
**Race**	Mexican American; Non-Hispanic White; Non-Hispanic Bla
	Other Hispanic; Other race
**Annual household income**	< 20000$, ⩾20000$
**Educational level**	Below high school; High school; Above high school;
**Body mass index**	Continuous
**Smoked at least 100 cigarettes in life**	No; Yes
**Had at least 12 alcohol drinks/year**	No; Yes
**Hypertension**	No; Yes
**Diabetes**	No; Yes
**Work physical activity**	Moderate; Vigorous; Other

**Recreational physical activity**	Moderate; Vigorous; Other
**Total energy intake (kcal/d)** [a]	Continuous
**Caffeine intake (mg/d)** [a]	Continuous

[a] the mean dietary intake through two 24-h dietary data.

Table S2. Baseline characteristics of participants by depressive symptoms, NHANES 2007–2018 (N=29050)

	Non-depressive symptoms (PHQ < 10)	Depressive symptoms (PHQ ≥ 10)	P value
Number of participants (%) [a]	26387 （90.83））	2663（9.07）	
Age	50 (35,64)	50 (37,62)	0.001
Age (year) [a]			<0.001
18-39	8481 (34.90)	806 (34.85)	
40-59	8631 (37.61)	1039 (42.55)	
≥60	9275 (27.49)	818 (22.60)	
Gender (%) [a]			<0.001
Male	13534 (50.64)	975 (35.72)	
Female	12853 (49.36)	1688 (64.28)	
Race/ethnicity (%) [a]			<0.001
Mexican American	3931 (8.33)	387 (7.81)	
Other Hispanic	2662 (5.51)	370 (8.01)	
Non-Hispanic White	11003 (67.75)	1131 (63.80)	
Non-Hispanic Black	5654 (10.86)	573 (13.21)	
Other races	3137 (7.54)	202 (7.17)	
Educational level (%) [a]			< 0.001
< high school	5955 (14.21)	847 (25.28)	
High school	6060 (22.86)	638 (26.66)	
> high school	14352 (62.93)	1075 (48.06)	
Household income (%) [a]			< 0.001
Under $20000	4865 (12.44)	968 (28.98)	
$20000 and over	20180 (87.56)	1531 (71.02)	
Body mass index	28.03(24.4,32.51)	30.06(25.29,35.36)	

Body mass index (%) [a]			< 0.001
< 18.5 kg/m^2	379 (1.39)	45 (2.08)	
18.5 to <25 kg/m^2	7095 (27.70)	573 (23.62)	
25 to <30 kg/m^2	8772 (33.19)	684 (25.64)	
≥30 kg/m^2	10141 (37.72)	1361 (48.66)	
Work activity (%) [a]			0.03
Vigorous	5323 (22.32)	508 (21.28)	
Moderate	5636 (24.17)	523 (21.68)	
Other	15420 (53.50)	1629 (57.03)	
Recreational activity (%) [a]			< 0.001
Vigorous	6106 (24.71)	275 (11.60)	
Moderate	6952 (28.51)	503 (22.07)	
Other	13326 (44.08)	1885 (66.32)	
Alcohol consumption (%) [a]	17510 (73.84)	1748 (70.26)	0.0013
Smoke at least 100 cigarettes in life (%) [a]	11455 (43.33)	1577 (61.08)	< 0.001
Diabetes (%) [a]	5080 (14.60)	734 (21.93)	< 0.001
Hypertension (%) [a]	14741 (51.03)	1654(58.03)	< 0.001
Ischemic heart disease (%) [a]	1742 (5.41)	344 (10.74)	< 0.001
Coronary heart disease (%) [a]	1010 (3.26)	182 (6.06)	< 0.001
Angina (%) [a]	587 (1.95)	151 (4.96)	< 0.001
Heart attack (%) [a]	1013(3.02)	201(6.23)	< 0.001
Central obesity (%) [a]	14418 (56.42)	1714(66.38)	< 0.001
Total energy (kcal/d) [b]	1906 (1465,2459.5)	1810.75(1353,2386.5)	< 0.001
Caffeine intake (mg/d) [b]	97.5 (29.198)	102 (30.221)	0.0056

Data are number of participants (weighted percentage) or medians (interquartile ranges).

PHQ, Patient Health Questionnaire; RDA, Recommended Dietary Allowance.

[a] Chi-square test was used to compare the percentage between participants with and without depression symptoms.

[b] Mann-Whitney U test was used to compare the difference between participants with and without depression symptoms.

Table S3. Weighted odds ratios (95% confidence intervals) of coronary heart disease stroke and anginas across depressive symptoms in model2, stratified by age gender BMI and waist, NHANES 2007–2018 (N = 29050)

	Ischemic heart disease	Coronary heart disease	Heart attack	Angina
**Age group**				
20-39 years old				
Depression	3.71(1.89,7.29) **	2.23(0.55,9.02)	3.81(1.72,8.49) **	3.66(1.09,12.30) *
Score	1.10(1.06,1.16) **	1.08(0.98,1.19)	1.11(1.05,1.17) **	1.12(1.03,1.20) **
40-59 years old				
Depression	2.37(1.55,3.61) **	2.22(1.18,4.15) *	2.30(1.37,3.85) **	3.55(2.01,6.27) **
Score	1.08(1.05,1.10) **	1.05(1.02,1.08) *	1.07(1.03,1.10) **	1.11(1.07,1.15) **
≥60 years old				
Depression	1.87(1.34,2.61) **	2.00(1.36,2.89) **	1.56(1.12,2.16) **	1.80(1.14,2.84) *
Score	1.06(1.03,1.08) **	1.06(1.04,1.09) **	1.06(1.03,1.08) **	1.06(1.03,1.09) **
**Gender group**				
Male				
Depression	2.17(1.60,2.94) **	2.02(1.36,3.68) **	2.14(1.52,3.03) **	2.49(1.49,4.17) **
Score	1.06(1.04,1.08) **	1.05(1.02,1.08) **	1.07(1.05,1.09) **	1.08(1.04,1.11) **
Female				
Depression	2.53(1.71,3.73) **	2.54(1.60,4.06) **	2.12(1.34,3.35) **	2.70(1.76,4.15) **
Score	1.09(1.06,1.12) **	1.09(1.06,1.12) **	1.07(1.04,1.11) **	1.10(1.06,1.13) **
Menopause				

Depression	2.26(0.77,6.67)	2.50(0.50,12.60)	5.44(0.98,30.23)	5.37(1.71,16.88) **
Score	1.09(1.02,1.17) *	1.07(0.95,1.21)	1.15(1.01,1.29) *	1.13(1.05,1.21) **
Non-menopause				
Depression	2.48(1.68,3.69) **	2.58(1.58,4.05) **	2.05(1.29,3.26) **	2.57(1.65,3.99) **
Score	1.09(1.06, 1.11) **	1.09(1.06,1.12) **	1.07(1.04,1.11) **	1.09(1.06,1.13) **
**Obesity group**				
BMI<30				
Depression	2.30(1.65,3.22) **	2.46(1.56,3.88) **	2.08(1.40,3.10) **	2.79(1.62,4.79) **
Score	1.07(1.05,1.09) **	1.08(1.05,1.10) **	1.07(1.05,1.10) **	1.08(1.05,1.12) **
BMI≥30				
Depression	2.49(1.84,3.37) **	2.12(1.35,3.34) **	2.23(1.62,3.07) **	2.55(1.67,3.88) **
Score	1.08(1.05,1.10) **	1.06(1.03,1.09) **	1.07(1.05,1.09) **	1.09(1.05,1.12) **
**Central obesity group**				
Waist<102/88cm for men/women				
Depression	2.05(1.33,3.17) **	2.97(1.51,5.86) **	1.74(1.01,3.00) **	2.92(1.25,6.84) *
Score	1.06(1.03,1.09) **	1.08(1.03,1.13) **	1.06(1.02,1.10) **	1.08(1.02,1.14) **
Waist≥ 102/88cm for men/women				
Depression	2.56(1.93,3.38) **	2.14(1.46,3.15) **	2.36(1.78,3.13) **	2.66(1.89,3.73) **
Score	1.08(1.06,1.10) **	1.07(1.04,1.09) **	1.08(1.06,1.10) **	1.09(1.06,1.12) **

a Calculated using binary logistic regression. Model 2 adjusted for age, gender, race/ethnicity, educational level, household income, caffeine intake, total energy intake, smoking, alcohol consumption, work activity, recreational activity diabetes, hypertension and BMI.

*P < 0.05; **P < 0.01

Table S4. Weighted odds ratios (95% confidence intervals) of ischemic heart disease, coronary heart disease, stroke and anginas across depressive symptoms in model 2, stratified by race, NHANES 2007–2018 (N = 29050)

	Ischemic heart disease	Coronary heart disease	Heart attack	Angina
**Race group**				
Mexican American				
Depression	2.35(1.25-4.42) **	1.75(0.82,3.74)	3.76(2.09,6.75) **	2.96(1.09,8.07) **
Score	1.09(1.05,1.13) **	1.07(1.01,1.14) *	1.11(1.07,1.15) **	1.11(1.05,1.17) **
Other Hispanic				
Depression	1.63(0.89,2.98)	2.01(0.80,5.11)	0.99(0.46,2.17)	2.09(0.93,4.68)
Score	1.06(1.03,1.10) **	1.08(1.02,1.13) **	1.04(1.01,1.08) *	1.07(1.02,1.23) *
Non-Hispanic White				
Depression	2.50(.179,3.48) **	2.42(1.57,3.74) **	2.22(1.57,3.14) **	2.76(1.80,4.24) **
Score	1.08(1.06,1.10) **	1.07(1.05,1.10) **	1.07(1.05,1.10) **	1.09(1.06,1.12) **
Non-Hispanic Black				
Depression	2.75(2.01,3.77) **	2.51(1.46,4.31) **	1.88(1.19,2.96) **	3.40(2.00,5.78) **
Score	1.07(1.05,1.10) **	1.07(1.03,1.11) **	1.06(1.02,1.09) **	1.09(1.05,1.14) **
Other Races				
Depression	2.09(0.94,4.65)	2.20(0.84, 5.78)	2.54(1.01,6.37) *	2.09(0.54,8.02)
Score	1.06(1.01,1.12) *	1.05(0.97,1.14)	1.08(1.01,1.14) *	1.07(0.99,1.16)

a Calculated using binary logistic regression. Model 2 adjusted for age, gender, race/ethnicity, educational level, household income, caffeine intake, total energy intake, smoking, alcohol consumption, work activity, recreational activity diabetes, hypertension and BMI.

*P < 0.05; **P < 0.01

TableS5 Synergic effect of depression and obesity on three types of ischemic heart disease incidence, NHANES 2007–2018 (N = 29050)

	RERI (95%CI)	P values	AP (95%CI)	P values
**Coronary heart disease**				
BMI	0.25(-1.38,1.88)	0.382	0.09(-0.42,0.59)	0.377
WAIST	0.15(-1.59,1.89)	0.432	0.05(-0.54,0.65)	0.431
**Heart attack**				
BMI	0.61(-0.59, 1.82)	0.158	0.21(-0.15,0.57)	0.129
WAIST	0.84(-0.28, 1.96)	0.070	0.31(0.00, 0.69)	0.049
**Angina**				
BMI	0.92(-0.97,2.81)	0.170	0.22(-0.19,0.64)	0.144
WAIST	0.72(-1.37,2.81)	0.251	0.21(-0.37,0.78)	0.242

The model adjusted for age, gender, race/ethnicity, educational level, household income, caffeine intake, total energy intake, smoking, alcohol consumption, work activity, recreational activity diabetes and hypertension.

Table S6 Odds ratios of ischemic heart disease coronary heart disease heart attack and anginas across depressive symptoms after multiple interpolation of missing depression data(N=33914)

	OR	SE	P-valve
Ischemic heart disease	2.32	0.07	<0.001
Coronary heart disease	2.11	0.08	<0.001
Angina	2.45	0.11	<0.001
Heart attack	2.16	0.09	<0.001

The model adjusted for age, gender, race/ethnicity, educational level, household income, caffeine intake, total energy intake, smoking, alcohol consumption, work activity, recreational activity, diabetes, hypertension and BMI)

Table S7. Weighted odds ratios (95% confidence intervals) of ischemic heart disease coronary heart disease heart attack and anginas across depressive symptoms in people who don't have cancer. NHANES 2007–2018 (N = 26126)

	OR	95%CI	P-valve
Ischemic heart disease	2.38	1.82-3.11	<0.001
Coronary heart disease	2.13	1.46-3.11	<0.001
Angina	2.70	1.87-3.88	<0.001
Heart attack	2.18	1.63-2.90	<0.001

aCalculated using binary logistic regression. The Model adjusted for age, gender, race/ethnicity, educational level, household income, caffeine intake, total energy intake, smoking, alcohol consumption, work activity, recreational activity, diabetes, hypertension and BMI.
